# Advances in Neural Networks and Hybrid-Metaheuristics: Theory, Algorithms, and Novel Engineering Applications

**DOI:** 10.1155/2016/3263612

**Published:** 2016-11-13

**Authors:** Marco A. Moreno-Armendáriz, Martin Hagan, Enrique Alba, José de Jesús Rubio, Carlos A. Cruz-Villar, Guillermo Leguizamón

**Affiliations:** ^1^Instituto Politécnico Nacional, Ciudad de México, Mexico; ^2^Oklahoma State University-Stillwater, Stillwater, OK, USA; ^3^Universidad de Málaga, Málaga, Spain; ^4^Cinvestav, Ciudad de México, Mexico; ^5^Universidad Nacional de San Luis, San Luis, Argentina

Among the many hot lines of modern research, hybridization stands out. Analyzing basic building blocks (ideas, algorithms, and procedures) and then building a new artifact (algorithm, machine, and tool) are in the core of Science. In this journal issue, we want to gather together some new and interesting ideas on neural networks and hybrid metaheuristics, two promising domains for building algorithms and techniques of higher efficiency and success.

In this special issue, a set of novel developments are presented. Out of the many submitted papers, just a few were accepted. Two main types of papers are published: the ones focusing on a hybrid methodology and the ones on applications.

As to novel methodologies, one of our papers develops active components of Scatter Search to improve cGA; the results show a significant improvement on the standard cGA. Another article in this issue presents the use of a novel metaheuristic to optimize a Convolutional Neural Network, leading to a net interesting improvement. In a different paper, we offer in this special issue a nice study of the calculus of the membership functions of a fuzzy system via Genetic Algorithms for video shot boundary detection: this work shows that the accuracy of the detection increases via the optimization process.

On the side of real applications, here we describe the ones included in this issue. The parallelization of a Back Propagation Neural Network using distributed computing technologies shows to be an effective way to improve the Neural Network performance in terms of efficiency. In another article, authors consider the safety and real-time working principles of intelligent vehicles: the Particle Swarm Optimization algorithm is used to calculate the heading angle and the path velocity for a robot.

This small collection of papers is just a little sample of many other developments done in the area, showing the relevance of this topic in terms of the significant improvements obtained with these techniques.

*Marco A. Moreno-Armendáriz* *Martin Hagan* *Enrique Alba* *José de Jesús Rubio* *Carlos A. Cruz-Villar* *Guillermo Leguizamón*

